# Annular Lichen Planus of the Penis Successfully Treated with Topical Tacrolimus 0.1% Ointment: A Case Report and Systematic Review of the Literature

**DOI:** 10.3390/life16030482

**Published:** 2026-03-16

**Authors:** Andrea D’Arino, Maria Concetta Fargnoli, Francesca Di Tullio, Carlo Cota, Paola Pasquini, Pasquale Frascione, Victor Desmond Mandel

**Affiliations:** 1 Oncologic and Preventive Dermatology Unit, San Gallicano Dermatological Institute-IRCCS, Via Chianesi 53, 00144 Rome, Italy; andrea.darino@ifo.it (A.D.); pasquale.frascione@ifo.it (P.F.); 2 San Gallicano Dermatological Institute-IRCCS, 00144 Rome, Italy; mariaconcetta.fargnoli@ifo.it; 3 STI/HIV Unit, San Gallicano Dermatological Institute-IRCCS, 00144 Rome, Italy; francesca.ditullio@ifo.it; 4 Dermatopathology Research Unit, San Gallicano Dermatological Institute-IRCCS, 00144 Rome, Italy; carlo.cota@ifo.it (C.C.); paola.pasquini@ifo.it (P.P.)

**Keywords:** lichen planus, annular lichen planus, genitalia, genital diseases, calcineurin inhibitors, tacrolimus

## Abstract

**Introduction:** Annular Lichen Planus (ALP) of the penis is a rare variant of genital lichen planus. Clinically, it is characterized by violaceous plaques with an annular configuration and central clearing. The diagnosis can be challenging, especially considering its rarity, and histopathological confirmation is often necessary to differentiate it from other annular dermatoses. **Methods:** We report a rare case of genital ALP in a male patient, while reviewing previously reported cases to provide a comprehensive overview of this rare condition. **Results:** A 63-year-old white male was referred to our outpatient department presenting with symptomatic annular erythematous-violaceous lesions on the glans penis. A diagnosis of genital ALP was clinically suspected and subsequently confirmed by histopathology. The patient was started on topical tacrolimus 0.1% ointment twice daily, achieving a complete clinical resolution after 6 consecutive weeks of treatment. No signs of local recurrence were observed after a one-year follow-up. We carried out a systematic review of the literature which identified a limited number of similar cases. **Conclusions**: This review underscores the importance of clinical suspicion and biopsy for accurate diagnosis and effective management of penile ALP. Topical tacrolimus 0.1% ointment is a safe and effective treatment for penile ALP and can be used as an alternative of corticosteroids.

## 1. Introduction

Lichen Planus (LP) is an uncommon, chronic inflammatory dermatosis that can involve the skin, mucous membranes, hair, and nails [1]. Incidence estimates vary between studies and demonstrate considerable geographical heterogeneity with figures in the order of tens per 100,000 persons/year [2]. Clinically, cutaneous LP generally presents with planar, purple, polygonal, pruritic papules and plaques (known as the 6 Ps of LP) [3] which can be superficially characterized by a lace-like whitish pattern known as Wickham striae. Genital involvement is considered less frequent, involving 25% of LP cases [4]. In addition to typical cutaneous lesions of LP, it may present with pruritus, burning, dyspareunia, and significant quality-of-life impairment [5].

Within the spectrum of genital LP, the annular variant–annular lichen planus (ALP)-is an uncommon presentation. ALP, characterized by annular-shaped violaceous lesions, with central clearing, is particularly rare, with limited reports documenting its occurrence in the male genital area. Moreover, this pattern overlaps with other annular dermatoses such as tinea corporis, annular psoriasis, granuloma annulare, and secondary syphilis. Key clues favoring ALP include a violaceous hue, sharply demarcated borders, and possibly Wickham striae; however, these features may be subtle on genital skin, making histopathology frequently necessary [6,7]. Typical changes are a lichenoid interface dermatitis with basal vacuolar degeneration, a band-like lymphocytic infiltrate obscuring the dermoepidermal junction, hypergranulosis, and Civatte bodies, helping differentiate it from mimickers [6,7,8].

Here, we report a case of ALP of the glans penis successfully treated with topical tacrolimus 0.1% ointment, and we systematically reviewed published cases of penile ALP with the aim of summarizing clinical presentation, possible differentials, and reported treatment outcomes.

## 2. Case Report

**Figure 1:**
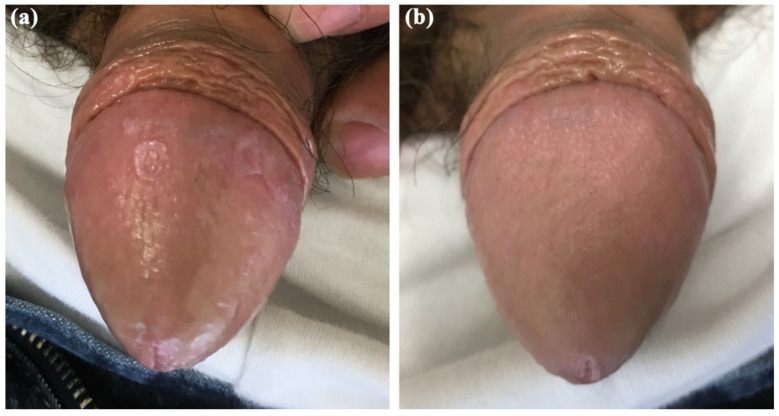
(**a**) Clinical appearance of the genital lesions. (**b**) Complete clinical resolution after 6 consecutive weeks of treatment with tacrolimus 0.1% ointment twice daily.

**Figure 2:**
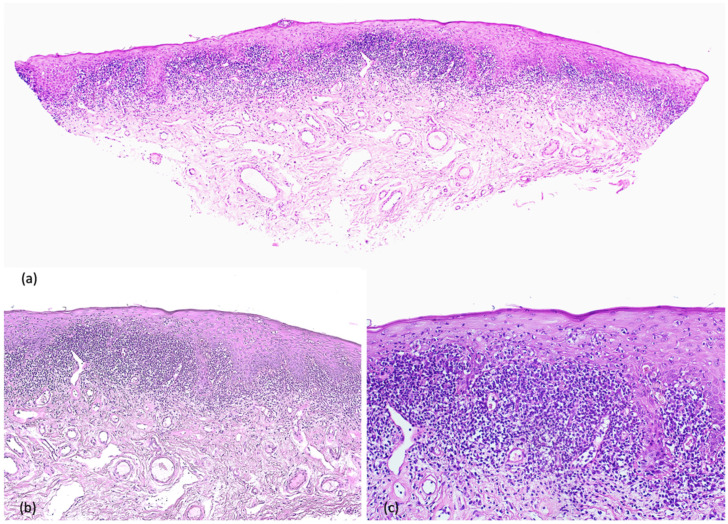
Histologic examination shows: (**a**) acanthotic epithelium with compact hyperkeratosis and a dense, band-like lymphocytic infiltrate at the dermoepidermal junction, partially obscuring the interface (HE 40×); (**b**,**c**) stratified squamous epithelium with mild acanthosis and surface orthokeratosis, without cytologic atypia. At the dermoepidermal junction, a dense, band-like lichenoid lymphocytic infiltrate is present, closely apposed to the basal layer and partially obscuring the interface. Basal cell vacuolar alteration and scattered apoptotic keratinocytes are present (HE 100×, 200×).

A 63-year-old white male was referred to our outpatient department in September 2024 for the appearance of annular erythematous-violaceous plaques with central clearing localized on the glans penis (Figure 1a). The lesions had been present for 6 months. The patient had not received any prior treatment for this condition. He reported persistent pruritus but had not attempted any self-treatment before seeking medical evaluation. His past medical history was notable for a history of bladder cancer, which had been treated with chemotherapy 15 years prior, and depressive–anxiety syndrome, managed with mirtazapine and clonazepam since 2009 and on stable dosage at the time of presentation. No new systemic medications had been initiated in the 10 years preceding lesion onset. The patient’s dermatological clinical history was otherwise unremarkable. A full body skin examination was conducted, and no other lesions were revealed on either the skin or oral mucosa. An incisional biopsy was performed to confirm the diagnosis, demonstrating histopathological findings consistent with the diagnosis of lichen planus, including a band-like lymphocytic infiltrate at the dermo–epidermal junction and basal vacuolar cell degeneration (Figure 2). Based on the clinical presentation and histopathological findings, the diagnosis of annular genital lichen planus was made. A drug-induced lichenoid eruption was considered among the differentials, considering the patient’s medication history and prior chemotherapy. However, the absence of new drug initiation and the stable long-term therapy, the localized genital presentation, and the histopathologic features supported ALP. The patient was started on topical tacrolimus 0.1% ointment twice daily with prompt improvement of the clinical symptoms and complete clinical resolution after 6 consecutive weeks of therapy (Figure 1b). A mild irritation was reported by the patient but decreased within the first few days of treatment. Topical tacrolimus was selected as first-line therapy as a steroid-sparing option for genital skin, aiming to minimize the risk of corticosteroid-induced atrophy and telangiectasia in a sensitive anatomic area. Calcineurin inhibitors are commonly used in genital LP when prolonged therapy is anticipated or when high-potency topical corticosteroids are not preferred. At one-year follow-up, no signs of local recurrence were observed.

## 3. Methods

### 3.1. Search Strategy

**Figure 3:**
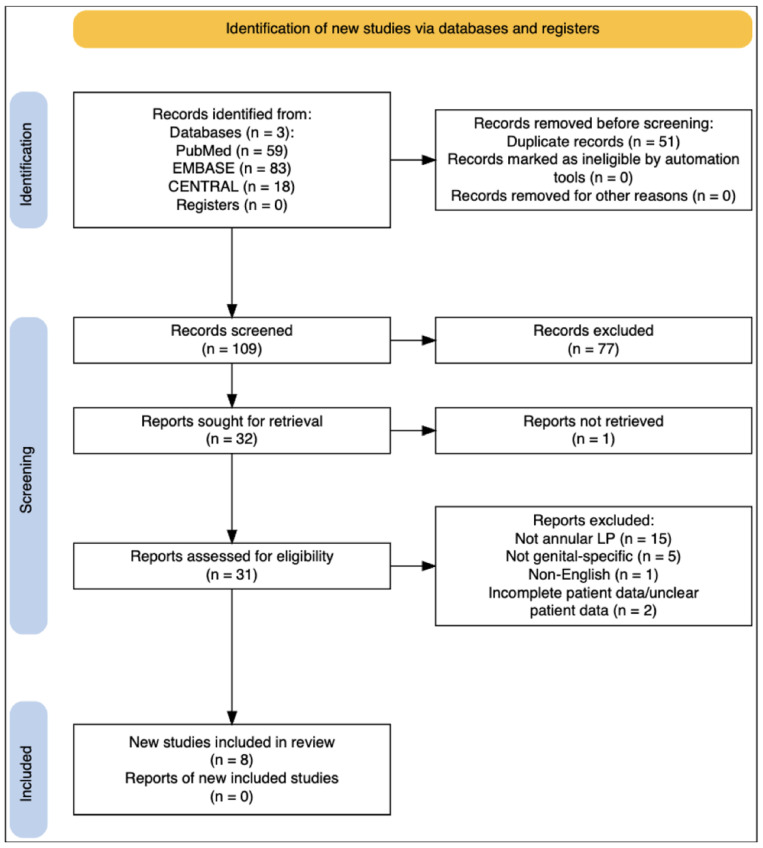
Systematic review PRISMA diagram.

We conducted a literature search on three databases: MEDLINE/PubMed (National Center for Biotechnology Information, NCBI), EMBASE (Ovid) and the Cochrane Central Register of Controlled Trials (CENTRAL) up to 30 September 2025. The search string included both free-text and/or Medical Subject Headings (MeSH and EMTREE) search of the following key search terms: “annular lichen planus” OR “genital lichen planus” in combination with (AND) “Genital Diseases”, “Penis”, “Vulva”, “Female Genitalia”, “HIV”. Sensitivity terms (e.g., vulva/female genitalia, HIV/immunocompromised) were used to capture potentially misindexed reports of genital annular LP; however, final eligibility criteria restricted inclusion to cases with penile involvement. The search method is fully described in the Supplementary S1, while the research strategy is outlined in Figure 3.

### 3.2. Eligibility Criteria

Only peer-reviewed case reports, case series, and observational studies specifically reporting ALP with penile involvement (glans, shaft, prepuce, coronal sulcus, or meatus), with sufficient clinical description and/or histopathologic confirmation, were included. No publication date limit was used. We excluded: (i) non-annular LP; (ii) genital LP without annular morphology; (iii) reports without adequate clinical detail or without clinicopathologic support; (iv) conference abstracts lacking full text; (v) non-English articles. Data on patient age, ALP characteristics, associated symptoms, treatment modalities, and extra-genital involvement were recorded from each included paper.

### 3.3. Study Selection and Data Extraction

Two authors (DAA and VDM) independently screened records and extracted data. Disagreements were resolved by consensus or by a third author (MCF) acting as referee.

### 3.4. Methodological Quality Appraisal

Methodological quality was assessed using the Joanna Briggs Institute (JBI) Critical Appraisal Checklists for Case Reports and Case Series. Each item was rated as *yes/no/unclear/not applicable*, and results are reported in Supplementary Tables S1 and S2.

## 4. Results and Discussion

The search identified 8 eligible publications on annular lichen planus with penile involvement (six case reports and two case series), which are summarized in Table 1. Methodological quality appraisal using the JBI critical appraisal tools rated case reports as low in 1/6 (3/8), moderate in 3/6 (5–6/8), and good in 2/6 (7/8), whereas both case series were low quality (2–3/10). The most frequent limitations across studies were incomplete follow-up reporting and non-standardized outcome assessment, which were considered when interpreting treatment response and recurrence. Considering the small number of publications and reported cases, we provide a narrative synthesis.

LP is an immune-mediated disorder with a still unknown etiology. Several pathogenetic mechanisms have been proposed, involving activated T cells, particularly CD8+ T cells, directed against basal keratinocytes. In clinical practice, it may affect the skin, mucous membranes, nails, scalp, and genitalia. Among the numerous variants reported, ALP is a rare morphological subtype that has been reported to present on the penis [17]. Patients typically present with asymptomatic or mildly pruritic annular plaques. The lesions appear as violaceous or brownish rings with a slightly raised, well-demarcated border and central clearing. ALP is most often interpreted as a morphologic pattern of LP rather than a distinct immunologic subtype [18]. Available literature does not clearly report unique immune signatures for ALP compared with classic cutaneous LP; histopathology is typically indistinguishable, supporting the view that annularity reflects lesion dynamics (centrifugal extension with relative central resolution) rather than a separate pathobiology [19].

One of the earliest reports, which dates back over a century, is that of Macleod et al., who documented the case of a 26-year-old man with “incomplete ringed lesions with an irregular border” localized on the penis [9]. Reports remained scarce until the late nineties when Barnette et al. and Matsuura et al. presented a total of three cases, two of the former and one of the latter [10,11]. These three cases present exclusively annular lesions localized on the penis, specifically on the glans. The earliest published cases of penile ALP primarily contributed to clinical recognition of the annular morphology on genital skin and highlighted the diagnostic risk of misclassification as fungal infection or other annular dermatoses. These seminal descriptions established that penile ALP could present with few ancillary clues and often require clinicopathologic correlation for diagnostic confidence.

More recent reports have continued to describe the penis, especially the glans, as a possible location of annular LP. The largest case series is presented by Reich et al., who included 20 ALP cases recorded during an 18-year period [12]. Among these, 25% of cases presented with an isolated genital presentation, similar to our case, but only three cases were localized on the glans penis. Only one patient showed extra-genital involvement on the eyelids. The lesions displayed the typical annular morphology, and almost all cases were asymptomatic. Similar annular morphology is reported by Badri et al., who detailed the case of a 29-year-old man with an isolated annular plaque on the glans penis, which had been present for one month [13]. Histopathology was necessary to confirm the diagnosis and showed typical LP findings such as vacuolar basal layer degeneration and a band-like lymphocytic infiltrate at the dermo–epidermal junction. In both these papers, given the limited extension of the disease, topical therapy with steroids was chosen as first-line treatment and resulted in improvement in most patients.

Morphology is also typical in the case presented by Isbary et al. [14]. However, they also report an association between ALP and genital lentigo. Nevertheless, given that this is the only reported case and the absence of controlled data, this relationship remains uncertain and should be regarded as hypothetical rather than clinically established.

An interesting case is that of Chakraborty et al., who described an HIV-positive patient who developed a rare, generalized form of ALP with genital involvement [15]. Histopathological findings were consistent with the diagnosis of ALP. This could suggest that immune dysregulation might influence the morphological presentation of LP.

Lastly, Natasatsekova et al. reported a single case of a 38-year-old male patient with genital and mucosal localization, treated with pimecrolimus 1%, with significant improvement [16].

Interestingly, no case reports were found for female genital ALP. Moreover, extra-genital involvement is rare in reported cases. While current evidence is insufficient to confirm ALP as a true distinct subtype with different pathogenetic mechanisms, its recurrent localization to genital skin and its characteristic annular morphology reported across cases could be cautiously interpreted as a recognizable pattern within the broader LP spectrum.

The annular variant of LP on the penis warrants a broad clinical differential diagnosis. Psoriasis, tinea corporis, granuloma annulare, and secondary syphilis might all present with similar annular lesions, underscoring the importance of skin biopsy and appropriate laboratory testing for a correct diagnosis.

Based on available literature, management of ALP of the penis should be tailored to the severity and extent of lesions and, as such, is generally carried out with topicals. No randomized clinical trials exist for this specific entity, and treatment is generally similar to broader LP management. Most of the papers report mid- or high-potency topical steroids as first-line therapy, which generally induce lesion regression. Of course, this comes with some limitations as the genital area differs in anatomy, tolerability, and risk profile of topical agents when compared to other body areas and steroid-sparing options can be clinically relevant on genital skin where long-term steroid adverse effects are of concern. Thus, topical calcineurin inhibitors are considered valid alternatives as they offer the advantage of long-term use with no risk for skin atrophy.

To the best of our knowledge, we did not identify prior reports specifically reporting topical tacrolimus 0.1% ointment for the treatment of penile ALP. Nevertheless, the use of calcineurin inhibitors has been reported in other LP variants and in classic genital LP.

A twice-daily application schedule is considered the preferred regimen for LP. A synthesis of controlled and open studies suggests that topical tacrolimus can achieve efficacy at least comparable to clobetasol 0.05% [14]. Local burning, prickling, itching and redness can be encountered with topical tacrolimus 0.1% ointment, but these adverse effects are usually transient and resolve after the first few days of treatment [14]. In addition, tacrolimus 0.1% ointment has higher efficacy and better tolerance than pimecrolimus 1% cream in some dermatological conditions like atopic dermatitis [14]. Therefore, the use of topical tacrolimus 0.1% ointment should be considered a valid treatment for penile ALP.

No articles reported the use of systemic immunosuppressive therapies for isolated penile ALP and these can probably be reserved for generalized severe cases.

We acknowledge several limitations of our study. The available evidence is limited to a small number of published reports and small case series, with heterogeneous reporting, potential publication bias, and limited follow-up; therefore, any inference regarding epidemiology and comparative treatment effectiveness should be interpreted cautiously and cannot be generalized. We also recognize that restricting inclusion to English-language full texts may have led to missed reports in other languages.

## 5. Conclusions

ALP is an uncommon LP variant that often involves the male genitalia and should be considered among the differential diagnosis of annular genital lesions. In our systematic review, all eligible publications exclusively described male patients. Clinicians should be aware of this rare entity when evaluating genital lesions in males, keeping a high index of suspicion for ALP. The list of clinical differentials is broad, including dermatophytosis, annular psoriasis, granuloma annulare, and secondary syphilis and obtaining a skin biopsy is recommended to confirm the diagnosis and to guide management. Topical tacrolimus 0.1% ointment could be considered as a steroid-sparing alternative on genital skin for the treatment of penile ALP, especially when long-term treatment is anticipated, cutaneous corticosteroid adverse effects (e.g., atrophy and telangiectasia) are of concern, or in cases with partial or unsatisfying response to corticosteroids.

## Figures and Tables

**Table 1:** Systematic review retrieved papers and patient characteristics.

Article	Age	Site	Symptoms	Histopathology (Description, If Available)	Treatment	Extra-Genital Localization
Macleod 1908 [9]	26	Penis shaft	Slight itching	No	Not reported	Yes, forearm
Barnette DI Jr. et al. 1993 [10]	36	Glans	Not reported	Yes (typical band-like lymphocytic infiltration and basal cell degeneration)	Not reported	No
	62	Glans	Not reported	Yes (focal acanthosis and basilar vacuolization with a band-like lymphocytic infiltrate)	Not reported	No
Matsuura C et al. 1998 [11]	66	Glans	Not reported	Yes (hyperkeratosis, focal hypergranulosis and vacuolation of basal cells with a band-like, dense cell infiltrate of lymphocytes)	Not reported	No
Reich HL et al. 2004 [12]	24	Penis (precise location not specified)	Pruritus	Yes (findings not specified)	The majority initially responded to treatment with mid- to high-potency topical steroids	Yes, eyelids
	28	Distal penile shaft	None	Yes (findings not specified)		No
	66	Glans	Burning, pruritus	No		No
	45	Glans	None	No		No
	39	Glans	None	No		No
	50	Scrotum	None	Yes (findings not specified)		No
Badri T et al. 2011 [13]	29	Glans	None	Yes (acanthosis, hyperorthokeratosis and Hypergranulosis, band-like lymphocytic infiltrate. Hydropic generation of basal layers, apoptotic bodies)	Topical clobetasol	No
Isbary G et al. 2014 [14]	37	Glans	Burning, pruritus	Yes (findings not specified)	Topical betamethasone, followed by pimecrolimus ointment	No
Chakraborty S et al. 2015 [15]	36	Scrotum	None	Yes–extra-genital (orthokeratotic hyperkeratosis, mild acanthosis, and effacement of the rete ridges with basal layer degeneration, colloid bodies, Max-Joseph’s canal, bandlike infiltrate of lymphohistiocytes along the dermo-epidermal junction with pigment incontinence, and disruption of dermal collagen)	High-potency topical steroid	Yes, generalized
Natasatsekova et al. 2017 [16]	38	Glans and shaft	None	Yes (findings not specified)	Topical pimecrolimus 1%	Yes, mucosal

## Data Availability

The data that support the findings of this study are available from the corresponding author upon reasonable request.

## Supplementary Materials

The following supporting information can be downloaded at https://www.mdpi.com/article/10.3390/life16030482/s1, Supplementary S1: the search method; Table S1: JBI Appraisal of the case reports included; Table S2: JBI Appraisal of the case series included.
