# Supplementary material for: Annular Lichen Planus of the Penis Successfully Treated with Topical Tacrolimus 0.1% Ointment: A Case Report and Systematic Review of the Literature
Source: Life (Basel). 2026 Mar 16;16(3):482. doi: 10.3390/life16030482 (PMC13028035; doi:10.3390/life16030482)
Supplement: Supplementary file 1 [file life-16-00482-s001.zip › life-4151902-supplementary/Supplementary Materials - Search strategy.pdf]

Supplementary S1

Search methods

The research has been done on three databases: MEDLINE/PubMed (National Center for Biotechnology Information, NCBI), EMBASE (Ovid) and the Cochrane Central Register of Controlled Trials (CENTRAL) until 16/04/2025.The search string contains free-text and/or Medical Subject Headings (MeSH and EMTREE) search of the following key search terms: "annular lichen planus" OR “genital lichen planus” in combination with (AND) “Genital Diseases”, “Penis”, “Vulva”, “Female Genitalia”, HIV.

Cochrane Central Register of Controlled Trials (CENTRAL)

search strategy

#1 ('annular lichen planus' OR 'genital lichen planus' OR "Generalized annular lichen planus" OR “anular atrophic lichen planus”):ti,ab,kw

#2 MeSH descriptor: [Genital Diseases] explode all trees

#3 ("Genital Disease" OR "Diseases, Genital" OR "Disease, Genital"):ti,ab,kw

#4 MeSH descriptor: [Penis] explode all trees

#5 ("Penis, Glans" OR "Glans Penis"):ti,ab,kw

#6 MeSH descriptor: [Genitalia] explode all trees

#7 ("Sex Organs, Accessory" OR "Organ, Accessory Sex" OR "Accessory Sex Organ" OR "Organs, Accessory Sex" OR "Accessory Sex Organs" OR "Sex Organ, Accessory" OR "System, Genital" OR "Genital Organ" OR "Genital" OR "Organs, Genital Systems" OR "Genital" OR "Organs, Reproductive Systems, Reproductive" OR "Reproductive System" OR "Genital Systems" OR "System, Reproductive" OR "Organ, Reproductive" OR "Reproductive Organs" OR "Reproductive Systems" OR "Organ, Genital" OR "Genitals" OR "Genital Organs" OR "Genital System" OR "Reproductive Organ"):ti,ab,kw

#8 MeSH descriptor: [Penile Diseases] explode all trees

#9 ("Penis Disease" OR "Penile Disease" OR "Disease, Penile" OR "Diseases, Penile" OR "Diseases, Penis" OR "Disease, Penis" OR "Penis Diseases"):ti,ab,kw

#10 ("Female Genitalia"):ti,ab,kw

#11 MeSH descriptor: [HIV] explode all trees

#12 ("Human Immunodeficiency Virus" OR AIDS OR HIV):ti,ab,kw

#13 MeSH descriptor: [Genitalia] explode all trees

#14 MeSH descriptor: [Genital Diseases, Female] explode all trees

#15 "Female Genital Diseases" OR "Gynecologic Disease" OR "Genital Disease, Female" OR "Female Genital Disease" OR "Gynecologic Diseases" OR "Diseases, Gynecologic" OR "Diseases, Female Genital"

#16 MeSH descriptor: [Vulva] explode all trees

#17 (Vulva*):ti,ab,kw

#18 MeSH descriptor: [Vulva] explode all trees

#19 #2 OR #3 OR #4 OR #5 OR #6 OR #7 OR #8 OR #9 OR #10 OR #11 OR #12 OR #13 OR #14 OR #15 OR #16 OR #17 OR #18

#20 #1 AND #19

EMBASE (Ovid)

search strategy

#1 'annular lichen planus'/exp OR 'annular lichen planus' OR 'genital lichen planus'/exp OR 'genital lichen planus': ab,ti

#2 'penis'/exp OR 'penis volume' OR 'phallus' OR 'penis': ab,ti

#3 'genital system disease'/exp OR 'disease of the reproductive system' OR 'disorder of the reproductive system' OR 'genital disease' OR 'genital diseases' OR 'genital disorder' OR 'genital system disorder' OR 'reproductive system disease' OR 'reproductive system disorder' OR 'genital system disease'):ab,ti

#4 'genital*': ab,ti

#5 #2 OR #3 OR #4

#6 #1 AND #5

MEDLINE/PubMed

search strategy

1 'annular lichen planus'[Title/Abstract] OR 'genital lichen planus'[Title/Abstract] OR "Generalized annular lichen planus"[Title/Abstract] OR “anular atrophic lichen planus”[Title/Abstract]

2 'Genital Diseases'[Mesh] OR 'Disease, Genital'[Title/Abstract] OR 'Diseases, Genital'[Title/Abstract] OR 'Genital Disease*'[Title/Abstract] OR 'Genital Disorder*'[Title/Abstract]

3 "Penis"[Mesh] OR "Penis"[Title/Abstract] OR "Glans Penis"[Title/Abstract] OR "Penis, Glans"[Title/Abstract]

4 "Genitalia"[Mesh] OR "Reproductive Organs"[Title/Abstract] OR "Organ, Reproductive"[Title/Abstract] OR "Organs, Reproductive"[Title/Abstract] OR "Reproductive Organ*"[Title/Abstract] OR "Reproductive System*"[Title/Abstract] OR "System, Reproductive"[Title/Abstract] OR "Systems, Reproductive"[Title/Abstract] OR "Genital System*"[Title/Abstract] OR "System, Genital"[Title/Abstract] OR "Genital*"[Title/Abstract] OR "Genital Organ*"[Title/Abstract] OR "Organ*, Genital"[Title/Abstract]

5 "Penile Diseases"[Mesh] OR “Disease, Penile"[Title/Abstract] OR "Diseases, Penile"[Title/Abstract] OR "Penile Disease"[Title/Abstract] OR "Penis Diseases"[Title/Abstract] OR "Disease, Penis"[Title/Abstract] OR "Diseases, Penis"[Title/Abstract] OR "Penis Disease"[Title/Abstract] OR Genital*[Title/Abstract] OR "Female Genitalia"[Title/Abstract]

6 "Acquired Immunodeficiency Syndrome"[Mesh] OR "Acquired Immunodeficiency Syndromes"[Title/Abstract] OR AIDS[Title/Abstract] OR "Acquired Immunodeficiency Syndrome"[Title/Abstract] OR HIV[Title/Abstract]

7 #2 OR #3 OR #4 OR #5 OR #6

8 #1 AND #7
