# Supplementary material for: Annular Lichen Planus of the Penis Successfully Treated with Topical Tacrolimus 0.1% Ointment: A Case Report and Systematic Review of the Literature
Source: Life (Basel). 2026 Mar 16;16(3):482. doi: 10.3390/life16030482 (PMC13028035; doi:10.3390/life16030482)
Supplement: Supplementary file 1 [file life-16-00482-s001.zip › life-4151902-supplementary/Supplementary Tables - JBI Appraisal of the studies included.pdf]

Table S1. Case Reports (JBI – 8 items).

Study	Patient description	Clinical history	Clinical examination	Diagnostic methods	Intervention described	Follow-up reported	Outcomes described	Conclusions supported	Total (0–8)	Quality
Macleod 1908 [9]	Yes	Yes	Yes	No	No	No	No	Unclear	3	Low
Matsuura C et al. 1998 [11]	Yes	Yes	Yes	Yes	No	No	No	Yes	5	Moderate
Badri T et al. 2011 [13]	Yes	No	Yes	Yes	Yes	No	Yes	Yes	6	Moderate
Isbary G et al. 2014 [14]	Yes	Yes	Yes	Yes	Yes	No	Yes	Unclear	6	Moderate
Chakraborty S et al. 2015 [15]	Yes	Yes	Yes	Yes	Yes	No	Yes	Yes	7	Good
Natasatsekova et al 2017 [16]	Yes	Yes	Yes	Yes	Yes	No	Yes	Yes	7	Good

Quality grading:

- 0–3 = Low
- 4–6 = Moderate
- 7–8 = Good

Table S2. Case Series (JBI – 10 items).

Study	Inclusion criteria	Recruitment method	Standardized measurement	Complete inclusion	Demographics reported	Clinical info reported	Intervention reported	Outcomes reported	Adequate follow-up	Appropriate analysis	Total (0–10)	Quality
Barnette DI Jr et al. 1993 [10]	No	No	No	No	Yes	Yes	No	No	No	No	2	Low
Reich HL et al. 2004 [12]	Yes	Unclear	Unclear	No	Yes	Yes	Unclear	Unclear	No	Unclear	3	Low

Quality grading:

- 0–4 = Low
- 5–7 = Moderate
- 8–10 = Good
